# Case Report: Shared decision-making in severe rheumatic mitral stenosis complicating pregnancy

**DOI:** 10.3389/fcvm.2026.1918698

**Published:** 2026-07-24

**Authors:** Luis Armando Velásquez Trujillo, Manuel Alejandro Hurtado Rivera, Víctor Rafael Bucheli Enríquez, Sebastián Ayala Zapata, Stephany Barbosa Balaguera, Álvaro Andrés Herrera Escandón

**Affiliations:** 1 Department of Internal Medicine, Universidad del Valle, Cali, Colombia; 2 Cardiovascular Surgery Research Group, Department of Cardiology, Clínica Sebastián de Belalcázar (Clínica Colsanitas S.A.), Cali, Colombia

**Keywords:** cardio-obstetrics, mitral valve replacement, multidisciplinary care, pregnancy, rheumatic mitral stenosis, shared decision-making, three-dimensional echocardiography

## Abstract

Severe rheumatic mitral stenosis (MS) is one of the highest-risk valvular lesions in pregnancy, and it becomes particularly hard to manage when the valve is unsuitable for percutaneous balloon mitral valvuloplasty (PBMV) and the patient declines termination. We describe a 20-year-old primigravida who presented at 23.5 weeks of gestation with progressive dyspnea, having deteriorated from New York Heart Association (NYHA) class I to class III. Multimodality echocardiography confirmed severe rheumatic MS, with a valve area of 0.5 cm^2^ by two-dimensional planimetry and 0.572 cm^2^ by three-dimensional transesophageal planimetry, a mean transmitral gradient of 15 mmHg, and a Wilkins score of 13, together with severe left atrial dilatation and pulmonary hypertension. PBMV was not feasible. After a shared decision-making process that respected the patient's refusal of termination, a multidisciplinary cardio-obstetric team provided a medical bridge aimed at fetal viability. Acute pulmonary edema at 28.1 weeks led to cesarean delivery of a 940 g neonate, and the patient underwent uncomplicated mechanical mitral valve replacement two weeks postpartum. The case shows how shared decision-making, multidisciplinary care, and the timing of surgery after delivery can be combined to protect both mother and child.

## Introduction

Rheumatic heart disease is still the leading cause of maternal cardiac death in low- and middle-income countries, and severe mitral stenosis (MS) is one of the most dangerous valvular lesions in pregnancy. The disease persists in these settings because untreated or recurrent group A streptococcal pharyngitis, overcrowding, poverty, and limited access to primary care and to secondary antibiotic prophylaxis allow rheumatic carditis to progress silently to chronic valve damage, so that young women often reach reproductive age with advanced, previously undiagnosed mitral stenosis. The fixed obstruction to left ventricular filling tolerates poorly the rise in plasma volume, heart rate, and cardiac output of the second and third trimesters, and it can precipitate decompensation even in women who were previously asymptomatic (1). Management becomes especially difficult when the valve anatomy rules out percutaneous balloon mitral valvuloplasty (PBMV) and the patient declines pregnancy termination, a situation that current guidelines address only in part.

We describe the management of a young primigravida with severe rheumatic MS and a Wilkins score of 13. The case brings together three decisions; respecting an autonomous refusal of termination that ran counter to the highest-risk medical recommendation; sustaining the pregnancy with medical therapy toward fetal viability when PBMV was contraindicated; and postponing definitive valve surgery until after delivery to spare the fetus exposure to cardiopulmonary bypass. We illustrate the diagnosis with multimodality echocardiography, including three-dimensional (3D) transesophageal planimetry, and we frame the decisions around respect for maternal autonomy in a setting where rheumatic disease.

## Case presentation

A 20-year-old primigravida (gravida 1) presented at 23.5 weeks of gestation with progressive exertional dyspnea. Her functional class had worsened from New York Heart Association (NYHA) class I before pregnancy to class III at presentation. She had received no antenatal care before this consultation. She had no known cardiac disease, no history of rheumatic fever (probably underdiagnosed in her region because of limited access to care), and no prior cardiac procedures or pregnancies. There was no family history of rheumatic fever, congenital or valvular heart disease, and no relevant hereditary or genetic condition. She was a homemaker from a low-income area of Colombia, with limited formal education, poor and intermittent access to health services, and a fragile social support network, a psychosocial context that is itself a well-recognized driver of underdiagnosed and undertreated rheumatic heart disease. N-terminal pro-B-type natriuretic peptide (NT-proBNP) was 477 pg/mL, consistent with early cardiac decompensation.

On examination there was a diastolic rumble at the apex, grade 2 jugular venous distension, and bilateral lower-limb edema, suggesting heart failure of valvular origin. The combination of a diastolic rumble, left atrial enlargement, and high filling pressures in a young woman from a region with a high burden of rheumatic disease pointed to rheumatic MS. Peripartum cardiomyopathy was ruled out by the preserved left ventricular systolic function and the structural valve disease seen on echocardiography. Congenital, calcific, and drug induced causes of MS were unlikely given the patient's age, the valve morphology, and the epidemiological context.

**Figure 1:**
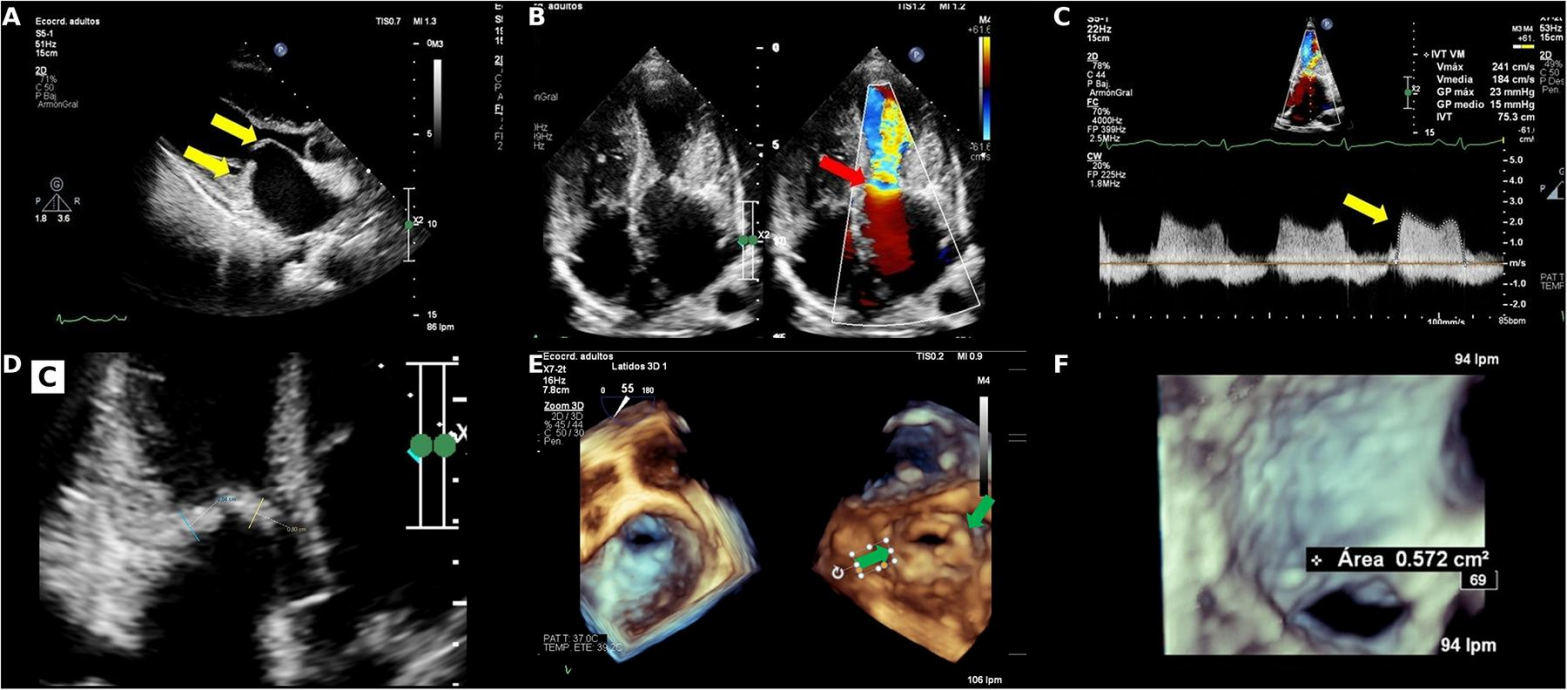
Diagnostic multimodality echocardiography at presentation. **(A)** Parasternal long-axis view showing diffuse mitral leaflet thickening with a hockey-stick deformity of the anterior leaflet (yellow arrows). **(B)** Apical four-chamber color Doppler showing a turbulent, high-velocity, mosaic diastolic jet through the narrowed orifice (red arrow), with severe biatrial enlargement. **(C)** Transmitral continuous-wave Doppler (yellow arrow) showing a peak velocity of 241 cm/s and a mean gradient of 15 mmHg. **(D)** Two-dimensional planimetry of the mitral orifice (0.84 by 0.80 cm), with diffuse leaflet thickening and commissural fusion. **(E)** Three-dimensional transesophageal echocardiography (3D TEE), en-face view from the left atrial side, showing the fish-mouth commissural fusion of the stenotic orifice (green arrows). **(F)** 3D TEE direct planimetry of the mitral orifice, giving an effective orifice area of 0.572 cm^2^.

Multimodality echocardiography, comprising a transthoracic study and three-dimensional transesophageal echocardiography (3D TEE), confirmed severe rheumatic mitral stenosis; the detailed diagnostic are shown in Figure 1. Marked commissural calcification was present, together with heavy leaflet thickening and calcification, diffuse commissural fusion, and extensive subvalvular involvement, so that all four components of the Wilkins score contributed to a total of 13, indicating anatomy that was clearly unfavorable for percutaneous intervention. The presence of significant commissural calcification, rather than the high score alone, was decisive, because balloon commissurotomy of a calcified commissure carries a high risk of severe mitral regurgitation and procedural failure. The left atrium was severely dilated (volume index 55 mL/m^2^), with no thrombus at this moment. Estimated pulmonary artery systolic pressure was 49mmHg (peak tricuspid regurgitation velocity 3.3 m/s), and left ventricular ejection fraction was preserved (55%–60%). The patient stayed in sinus rhythm and atrial fibrillation was never documented.

Because the maternal and fetal risk was very high, the team offered voluntary termination on maternal grounds, but the patient declined. Respecting her decision, which reflects a right to reproductive self-determination protected under Colombian law, a multidisciplinary cardio-obstetric team ((clinic cardiology, cardiovascular surgery, obstetrics, anesthesia and neonatology) agreed on a conservative plan aimed at reaching fetal viability. The decision was reached through several multidisciplinary boards held across the admission. The very high maternal mortality of continuing the pregnancy, the poor anatomical suitability for any percutaneous intervention, and the neonatal prognosis at successive gestational ages were explained to the patient directly and in plain language over repeated sessions. No relatives took part in these discussions, since the patient had a limited and unstable support network, so particular care was taken to confirm that her repeatedly expressed choice was informed, voluntary, and stable. This included close surveillance in a high-risk obstetric unit, rate control with oral metoprolol to keep the heart rate below 70 beats per minute, anticoagulation, diuretics, and serial monitoring with NT-proBNP every 72 h and echocardiography every week. Serial NT-proBNP was chosen because natriuretic peptides tend to rise before overt clinical deterioration in pregnant women with heart disease and are recommended by current guidelines as an objective marker for risk stratification and follow-up, so that a rising trend could act as an early warning of failing compensation and prompt escalation of therapy or delivery (1). Anticoagulation was indicated by the severe left atrial dilatation (volume index 55 mL/m^2^) and the resulting low-flow, prothrombotic state, even though the patient stayed in sinus rhythm and had no atrial fibrillation or demonstrable left atrial thrombus. It was started with therapeutic low-molecular-weight heparin; anti-Xa levels could not be measured because the assay was not available at our center, which is a recognized limitation of heparin dosing in this setting. At 29 weeks, once the second trimester was well advanced and the period of greatest risk of warfarin embryopathy, which is largely confined to the first trimester, had passed, treatment was changed to warfarin at a stable dose of 5 mg per day with a target INR of 3.0 (range 2.5–3.5). A daily maintenance dose at or below 5 mg is associated with a substantially lower risk of fetal complications, and a vitamin K antagonist offered more reliable and more easily monitored anticoagulation than weight-adjusted heparin without anti-Xa control. The patient and the team accepted the small residual fetal risk in exchange for more secure thromboembolic protection during the remainder of the pregnancy. PBMV was considered but ruled out because of the unfavorable anatomy (Wilkins score 13). The aim was to prolong the pregnancy to about 28 weeks to improve neonatal survival. Obstetric surveillance during this period showed adequate fetal growth and a reassuring fetal heart rate. With diuretics and beta-blockade, NT-proBNP fell from 477 to between 180 and 390pg/mL, and serial echocardiograms showed preserved systolic function and stable hemodynamics.

At 28.1 weeks the patient developed acute pulmonary edema, that did not respond to intensified medical therapy, which marked the failure of conservative management and forced delivery. The obstetric team chose cesarean delivery. In fixed severe MS with acute decompensation, cesarean allowed a prompt and controlled delivery under general anesthesia with continuous invasive cardiovascular monitoring, and it avoided the repeated rises in heart rate, the autotransfusion and preload shifts of uterine contractions, and the valsalva effort of the second stage of labor, all of which acutely worsen the transmitral gradient and pulmonary congestion. The cesarean was performed under general anesthesia, chosen to secure the airway, control heart rate, and avoid the abrupt sympathetic and preload changes that a neuraxial block can cause in a fixed obstructive lesion. In addition, to standard monitoring, invasive arterial pressure was recorded continuously, with the aim of keeping the heart rate low and preload and afterload stable. No vasoactive drugs were required, since the patient remained hemodynamically stable throughout. To avoid the tachycardia and vasodilatation that a bolus of oxytocin can provoke, the uterotonic was given only as a slow infusion, at 2.5IU per hour for the first five minutes and 7.5 IU per hour thereafter, titrated to uterine tone. The extreme prematurity and the poor conditions for a rapid vaginal delivery also supported this choice. A live male neonate weighing 940 g was delivered. He was admitted to the neonatal intensive care unit for extreme prematurity, needed mechanical ventilation, and stayed about one month, with favorable progress. At the most recent follow-up, at eighteen months of corrected age, the infant was thriving, with growth and neurodevelopment appropriate for age and no cardiac, respiratory, or neurological sequelae.

**Figure 2:**
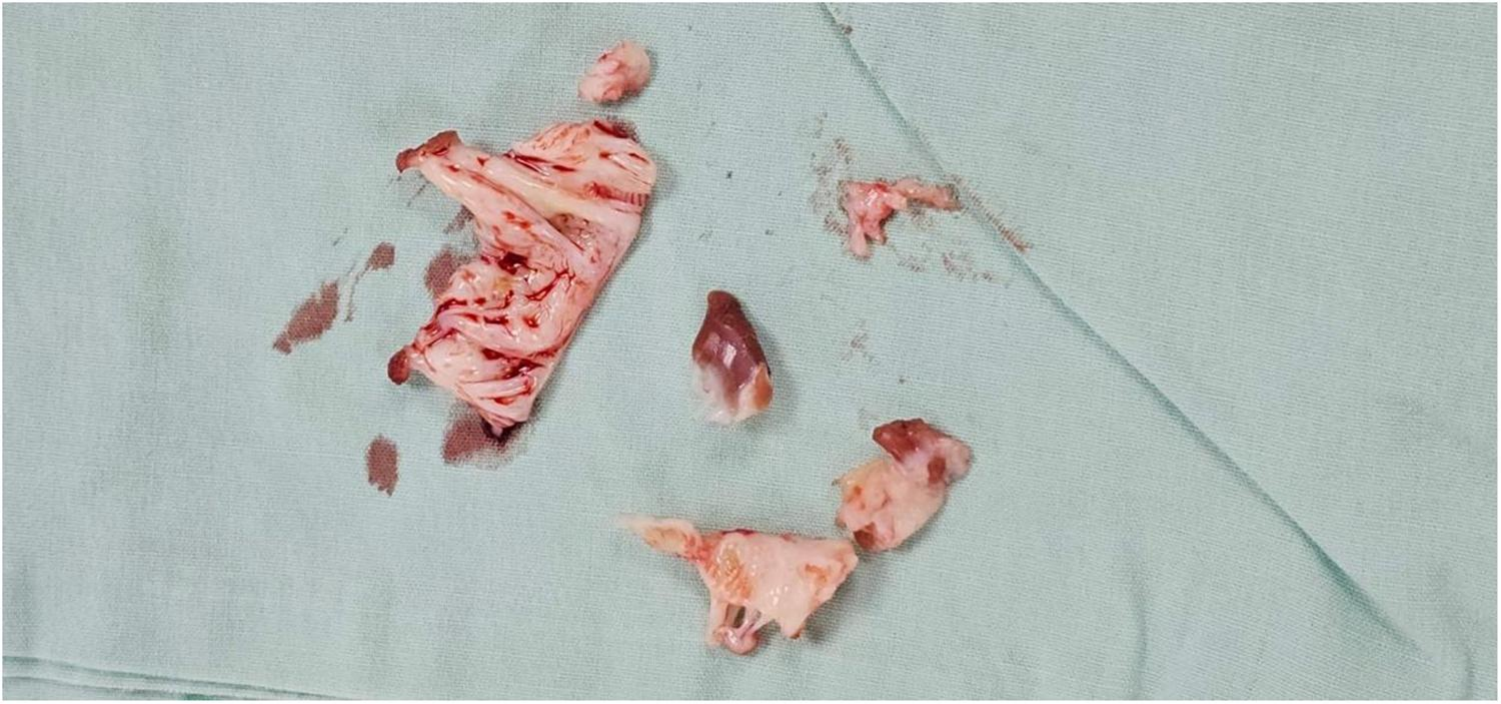
Intraoperative photograph of the explanted native mitral valve, showing severe leaflet thickening, diffuse commissural fusion, and advanced rheumatic fibrosis, which confirmed the unfavorable wilkins score that ruled out percutaneous balloon mitral valvuloplasty.

Two weeks postpartum, once she was hemodynamically stable, the patient underwent elective mitral valve replacement with a 27 mm mechanical prosthesis. The operative findings confirmed severely thickened, fused leaflets typical of advanced rheumatic disease (Figure 2). There were no complications. Postoperative TEE showed a normally functioning prosthesis with adequate leaflet motion (peak velocity 21.8 cm/s, gradient 0 mmHg) and no significant residual gradient, and left ventricular ejection fraction was preserved at 55%. Anticoagulation for the mechanical prosthesis was managed by overlapping low-molecular-weight heparin with warfarin, with daily INR measurement and dose adjustment until the target range of 2.5–3.5 was reached. She was discharged in stable condition on warfarin, antibiotic prophylaxis for rheumatic carditis, and outpatient follow-up with cardiology and obstetrics. At discharge her dyspnea had resolved and she had returned to NYHA class I. A timeline of the clinical course, from presentation to postpartum valve replacement, is summarized in Table 1.

**Table 1:** Timeline of the clinical course, from presentation with severe rheumatic mitral stenosis to postpartum mechanical mitral valve replacement.

Timepoint	Clinical status and key findings	Management and outcome
23.5 weeks (presentation)	Severe rheumatic MS, NYHA class III, early decompensation. MV area 0.5 cm^2^ (2D) and 0.572 cm^2^ (3D TEE), mean gradient 15 mmHg, Wilkins score 13 with commissural calcification, PASP 49 mmHg, sinus rhythm, NT-proBNP 477 pg/mL.	Admission to high-risk obstetric unit. Multidisciplinary board; voluntary termination offered and declined; PBMV ruled out for unfavorable anatomy.
Shared decision	Patient declined termination; choice confirmed as informed, voluntary, and stable. No relatives available.	Reproductive autonomy respected under Colombian law. Viability-guided medical bridge agreed by the cardio-obstetric team.
23.5 to 28 weeks (medical bridge)	Sinus rhythm maintained, stable hemodynamics, adequate fetal growth. NT-proBNP fell to 180 to 390 pg/mL.	Oral metoprolol (heart rate below 70 bpm), diuretics; LMWH then warfarin 5 mg (target INR 3.0) from 29 weeks. NT-proBNP every 72 h, weekly echocardiography.
28.1 weeks	Acute pulmonary edema refractory to intensified medical therapy.	Urgent cesarean under general anesthesia with invasive arterial monitoring, no vasoactive drugs, slow oxytocin infusion. Live male neonate 940 g admitted to NICU.
2 weeks postpartum	Hemodynamically stable.	Elective mechanical mitral valve replacement (27 mm), uncomplicated. Postoperative prosthesis peak velocity 21.8 cm/s, gradient 0 mmHg, LVEF 55%.
Discharge and follow-up	NYHA class I, dyspnea resolved.	Warfarin and prophylaxis for rheumatic carditis; cardiology and obstetric follow-up. Neonate thriving at 18 months with appropriate neurodevelopment.

LMWH, low-molecular-weight heparin; LVEF, left ventricular ejection fraction; MS, mitral stenosis; MV, mitral valve; NICU, neonatal intensive care unit; NYHA, New York Heart Association; PASP, pulmonary artery systolic pressure; PBMV, percutaneous balloon mitral valvuloplasty; 3D TEE, three-dimensional transesophageal echocardiography.

## Discussion

Severe rheumatic MS in pregnancy is one of the highest-risk situations in maternal cardiology. This patient met criteria for very high maternal risk from the start, which justified the initial recommendation of termination, in keeping with the modified World Health Organization class IV risk assigned by current guidelines (1, 7). Her refusal required the team to work within a shared decision-making framework, in line with current international guidelines that favor individualized care in specialized centers when reasonable hemodynamic stability can be achieved (1, 2).

Setting a realistic target for fetal viability was a central problem. The limit of viability is generally placed at around 24 weeks, and neonatal survival improves substantially after 26–28 weeks, so aiming for 28 weeks balanced acceptable maternal risk against meaningful benefit for the newborn (4, 5). Data from the ROPAC registry show that about half of women with severe rheumatic MS develop heart failure during pregnancy, with prepregnancy functional class and stenosis severity as the main predictors of poor maternal outcomes (3).

PBMV is the preferred intervention in pregnancy for women with favorable valve anatomy, but the benefit does not extend to those with a Wilkins score of 11 or more, severe subvalvular disease, or marked commissural calcification, in whom it carries a higher risk of severe mitral regurgitation and procedural failure (6, 7, 12). In this patient the marked commissural calcification, in addition to a Wilkins score of 13, ruled out PBMV, because balloon dilatation of a calcified commissure would have carried a high risk of severe acute mitral regurgitation, and left a medical bridge as the only way to gain time. Anticoagulation was given despite the absence of atrial fibrillation, because the severe left atrial dilatation and the low-flow state favor thromboembolism. Low-molecular-weight heparin was used first, which limits fetal exposure to vitamin K antagonists, and warfarin adjusted by INR was introduced later (2, 7, 12).

Pulmonary hypertension was a very important modifier of maternal risk in this case. The estimated systolic pulmonary artery pressure in echocardiography of 49mmHg reflected chronic transmission of the elevated left atrial pressure and identified a circulation with little reserve, in which any further rise in heart rate or in circulating volume could precipitate right ventricular strain and pulmonary edema. It therefore reinforced the very high risk category, argued against any attempt at intervention during pregnancy, and shaped the conservative plan, with strict rate control, careful diuresis, and a low threshold for delivery once congestion became refractory. The acute pulmonary edema at 28.1 weeks was the clinical expression of this limited reserve.

Acute pulmonary edema at 28.1 weeks clearly marked the failure of medical management and was a definite trigger for delivery, in keeping with guideline recommendations (1, 2). Deferring cardiac surgery to the postpartum period was a deliberate choice. Cardiac surgery with cardiopulmonary bypass during pregnancy carries an estimated fetal mortality of 10%–30%, whereas deferral lowers this risk substantially without a comparable rise in maternal mortality (8, 9). The early postpartum period is itself high-risk because of the sudden rise in preload after delivery of the placenta, so close monitoring during the first 24–48 h is essential (10). Performing the replacement two weeks postpartum, once the patient was stable, avoided the instability of a combined procedure and still corrected the lesion before discharge. Long-term data confirm that women with rheumatic MS who go through a complicated pregnancy are very likely to need definitive valve surgery in the following years (11).

These decisions can be placed alongside the few comparable reports in the literature. Eng-Frost and colleagues described a woman with severe rheumatic MS who remained asymptomatic throughout pregnancy and in whom percutaneous valvuloplasty was safely deferred until after delivery, a course that is reasonable only while the patient stays in a low functional class (13). Batra and colleagues reported a young woman with severe rheumatic MS, a Wilkins score of 12, and severe pulmonary hypertension in whom, as here, the anatomy was not favorable for balloon valvuloplasty; that team used mechanical circulatory support and proceeded to cesarean delivery followed by valve surgery (14). Our patient differs from the first in that she became symptomatic and reached NYHA class III, which removed watchful waiting as an option, and from the second in that she was managed without mechanical support, with a purely medical bridge to viability and a valve replacement deliberately deferred to the stable postpartum period. Taken together, these reports suggest that when balloon valvuloplasty is anatomically unfeasible, the choice between continued medical therapy and surgery during pregnancy should be driven by functional class, hemodynamic reserve, and the proximity of a viable gestational age, rather than by a fixed protocol.

The main contribution of this report is to show how three decisions can be combined into one coherent and reproducible cardio-obstetric pathway (Central Illustration): respecting an autonomous refusal of termination, providing a viability-guided medical bridge when PBMV is contraindicated, and deferring definitive surgery to the postpartum period. This is directly relevant to settings where rheumatic disease is still common.

**CENTRAL ILLUSTRATION:**
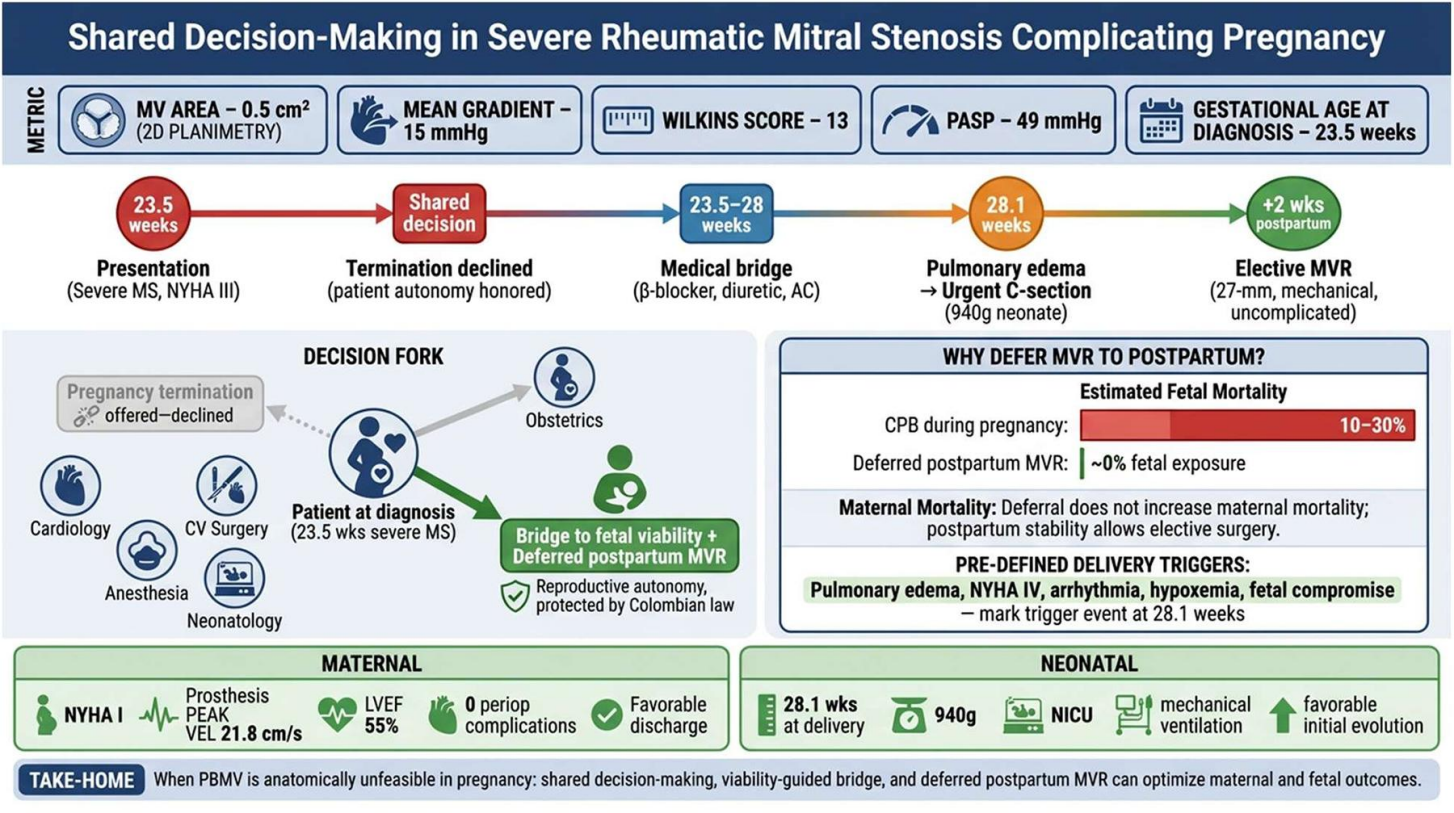
Visual summary of the case. The top band shows the five key diagnostic indices at presentation (mitral valve area by 2D planimetry, mean transmitral gradient, Wilkins score, pulmonary artery systolic pressure, and gestational age). The timeline marks five points: presentation at 23.5 weeks; the shared decision after the patient declined termination; the medical bridge (beta-blocker, diuretic, anticoagulation); acute pulmonary edema at 28.1 weeks leading to cesarean delivery of a 940 g neonate; and elective mitral valve replacement two weeks postpartum. The decision-fork panel shows the divergence between the offered (declined) and chosen pathways and the input of the multidisciplinary team. The adjacent panel compares the estimated fetal mortality of cardiopulmonary bypass during pregnancy (10 to 30%) with that of postpartum surgery (about 0% fetal exposure) and lists the predefined triggers for delivery. The lower strips summarize the favorable maternal and neonatal outcomes.

### Limitations

This is a single case, so the findings cannot be generalized, and the best timing for cesarean delivery and for definitive valve surgery needs confirmation in larger series. Follow-up beyond discharge is still relatively short. Neonatal progress has been favorable up to eighteen months of corrected age, but longer term neurodevelopment cannot yet be judged with certainty, and the stability of anticoagulation with the mechanical prosthesis and the outcome of contraceptive counseling continue to be followed. The care described took place in a specific cultural and legal context and in a tertiary academic referral center, so it may be harder to reproduce where comparable multidisciplinary resources are lacking. Finally, the absence of cardiac magnetic resonance imaging and invasive hemodynamic study reflects everyday practice but limits the detail of the preoperative assessment.

## Conclusion

This case shows how demanding the management of severe rheumatic MS in pregnancy can be, and it highlights the value of shared decision-making, multidisciplinary cardio-obstetric care, and sensible timing of surgery after delivery. Recognizing the failure of conservative treatment, setting a realistic target for fetal viability, and postponing definitive surgery until after delivery were the decisions that produced good outcomes for both mother and child. More broadly, this case supports individualized, multidisciplinary management as the standard of care for pregnant women with severe rheumatic mitral stenosis, particularly in low- and middle-income settings where the disease remains common, and where respect for maternal autonomy, careful timing of delivery, and coordinated cardio-obstetric follow-up can together turn a very high-risk pregnancy into a survivable one for both mother and child.

## Patient perspective

After being informed in detail of the maternal risks, the patient declined termination. She expressed a clear and repeatedly confirmed wish to continue the pregnancy, and she took an active part in the discussions that shaped the surveillance plan and the timing of delivery and surgery. At discharge she said she was satisfied with having been an informed participant in the decisions that affected her own health and that of her child. A direct quotation is not included, to preserve de-identification.

## Data Availability

The original contributions presented in the study are included in the article/Supplementary Material, further inquiries can be directed to the corresponding author.
